# Lymphotoxin β Receptor Signaling Induces IL-8 Production in Human Bronchial Epithelial Cells

**DOI:** 10.1371/journal.pone.0114791

**Published:** 2014-12-11

**Authors:** Yu Mikami, Hirotaka Matsuzaki, Masafumi Horie, Satoshi Noguchi, Taisuke Jo, Osamu Narumoto, Tadashi Kohyama, Hajime Takizawa, Takahide Nagase, Yasuhiro Yamauchi

**Affiliations:** 1 Department of Respiratory Medicine, Graduate School of Medicine, The University of Tokyo, Tokyo, Japan; 2 Department of Internal medicine, Teikyo University Mizonokuchi hospital, Kanagawa, Japan; 3 Department of Respiratory Medicine, Kyorin University, Tokyo, Japan; National Jewish Health, United States of America

## Abstract

Asthma-related mortality has been decreasing due to inhaled corticosteroid use, but severe asthma remains a major clinical problem. One characteristic of severe asthma is resistance to steroid therapy, which is related to neutrophilic inflammation. Recently, the tumor necrosis factor superfamily member (TNFSF) 14/LIGHT has been recognized as a key mediator in severe asthmatic airway inflammation. However, the profiles and intracellular mechanisms of cytokine/chemokine production induced in cells by LIGHT are poorly understood. We aimed to elucidate the molecular mechanism of LIGHT-induced cytokine/chemokine production by bronchial epithelial cells. Human bronchial epithelial cells express lymphotoxin β receptor (LTβR), but not herpesvirus entry mediator, which are receptors for LIGHT. LIGHT induced various cytokines/chemokines, such as interleukin (IL)-6, oncostatin M, monocyte chemotactic protein-1, growth-regulated protein α and IL-8. Specific siRNA for LTβR attenuated IL-6 and IL-8 production by BEAS-2B and normal human bronchial epithelial cells. LIGHT activated intracellular signaling, such as mitogen-activated protein kinase and nuclear factor-κB (NF-κB) signaling. LIGHT also induced luciferase activity of NF-κB response element, but not of activator protein-1 or serum response element. Specific inhibitors of phosphorylation of extracellular signal-regulated kinase (Erk) and that of inhibitor κB attenuated IL-8 production, suggesting that LIGHT-LTβR signaling induces IL-8 production via the Erk and NF-κB pathways. LIGHT, via LTβR signaling, may contribute to exacerbation of airway neutrophilic inflammation through cytokine and chemokine production by bronchial epithelial cells.

## Introduction

Bronchial asthma (BA) is a chronic airway inflammatory disease characterized by varying degrees of bronchial obstruction, airway hyperresponsiveness and airway remodeling. The standard therapy for asthma is administration of inhaled corticosteroids (ICS). A response to ICS predicts a good outcome [1].

The airway inflammation of BA is generally thought to be eosinophilic inflammation induced by T-helper lymphocytes following production of various levels of cytokines and chemokines by inflammatory cells [2]. Corticosteroids are generally effective for this airway inflammation [3]. However, the airway inflammation in some asthmatics resists corticosteroids and is considered to be severe/refractory asthma. Severe asthma is defined as asthma that requires treatment with a high-dose ICS plus a second controller and/or systemic corticosteroid to prevent it from becoming “uncontrolled”, or asthma that remains “uncontrolled” despite this therapy [4].

Because the number of neutrophils in sputum is increased in severe asthma compared to mild and moderate asthma [5] and is negatively associated with lung function [6], it is thought that neutrophils play a key role in the pathogenesis of severe asthma. Moreover, interleukin (IL)-8, a major chemoattractant of neutrophils [7], is also increased in severe asthmatic airways [8]. Corticosteroids generally decrease airway eosinophils and are, as noted above, effective in treating eosinophilic inflammation (3), but neutrophils induce steroid-resistant inflammation, which leads to airway remodeling that consequently exacerbates the morbidity of asthma [9], [10].

The tumor necrosis factor superfamily member (TNFSF) 14/LIGHT (homologous to lymphotoxins, exhibits inducible expression and competes with HSV glycoprotein D for herpesvirus entry mediator (HVEM), a receptor expressed by T lymphocytes [11]) is a type II membrane protein. LIGHT is produced by activated T cells and can bind to lymphotoxin β receptor (LTβR) and HVEM, both of which belong to the TNF receptor superfamily [12]. It is known that LIGHT-HVEM signaling regulates T-cell proliferation [13], while LIGHT-LTβR signaling induces apoptosis of cancer cells [14] and organization and maintenance of lymphoid structures [15]. Recently, LIGHT has been implicated in the pathogenesis of such inflammatory diseases as rheumatoid arthritis and inflammatory bowel disease [16], [17]. We have already reported that LIGHT contributes to the pathogenesis of airway fibrosis through enhancement of epithelial mesenchymal transition [18]. Doherty et al. [19] showed that LIGHT is expressed on lung inflammatory cells after allergen exposure and that blockade of LIGHT suppresses expression of transforming growth factor (TGF)-β and IL-13 in the lung. TGF-β and IL-13 are cytokines that are implicated in airway remodeling. Moreover, a pharmacological inhibitor of LTβR reduced smooth muscle hyperplasia and airway hyperresponsiveness in house dust mite-induced mouse models of chronic asthma [19].

In clinical practice, LIGHT levels in the sputum of asthma patients were negatively associated with lung function, suggesting that LIGHT is associated with asthma severity [20]. Another study showed that LIGHT contributed to synovial inflammation and neutrophil accumulation via IL-8 production by fibroblasts [16]. Taken together, LIGHT might induce neutrophilic inflammation via IL-8 production. However, possible effects of LIGHT on IL-8 production by bronchial epithelial cells have not been studied, and the mechanism of IL-8 production by cells has not been fully elucidated.

Therefore, we first evaluated the cytokine profile, especially inflammatory cytokines such as IL-8 and IL-6, of bronchial epithelial cells exposed to LIGHT. We then investigated the mechanisms of LIGHT-induced IL-8 production by elucidating the intracellular signaling.

## Materials and Methods

### Reagents

Recombinant human TNFSF14/LIGHT was purchased from R&D Systems Inc. (Minneapolis, MN) and dissolved in ddH2O. A selective mitogen-activated protein kinase (MAPK)/extracellular signal-regulated kinase (ERK) kinase (MEK) 1/2 inhibitor, U0126 (10 µM; Cell Signaling Technology, Beverly MA); a selective c-jun N-terminal kinase (JNK)-1, -2 and -3 inhibitor, SP600125 (25 µM; Cell Signaling Technology); a selective inhibitor of p38 MAPK, SB203580 (25 µM; Cell Signaling Technology); an irreversible inhibitor of inhibitor κB (IκB) kinase α and phosphorylation of cytokine-inducible IκBα, BAY11-7082 (10 µM; Sigma-Aldrich, St. Louis, MO), and phorbol 12-myristate 13-acetate (PMA, 50 mg/ml; Sigma-Aldrich), were each dissolved in dimethyl sulfoxide (DMSO).

### Cells and cell culture

BEAS-2B cells, an immortalized cell line established from normal human bronchial epithelium obtained by autopsy of non-cancerous individuals and widely used to study the functions of lung bronchial epithelial cells [21], were purchased from the American Type Culture Collection (Manassas, VA). Primary normal human bronchial epithelial cells (NHBE) were purchased from TAKARA BIO INC. (Tokyo, Japan). The cells were cultured in serum-free epithelial growth medium (BEGM; Cambrex, Walkersville, MD) supplemented with Bullet Kit (Cambrex) to contain 0.5 ng/ml human recombinant epidermal growth factor, 0.5 µg/ml hydrocortisone, 10 µg/ml transferrin, 0.5 µg/ml epinephrine, 5 µg/ml insulin, 50 µg/ml bovine pituitary extract, 0.1 ng/ml retinoic acid, 6.5 ng/ml triiodothyronine, 50 µg/ml gentamicin and 0.1 ng/ml amphotericin B at 37°C in a humidified 5% CO_2_ atmosphere. THP-1 cells, a human monocytic cell line, were purchased from the American Type Culture Collection. Cells were cultured in 10-cm culture dishes in RPMI 1640 medium supplemented with 10% fetal bovine serum at 37°C in a humidified 5% CO_2_ atmosphere.

### Cell surface receptor analysis

Cell surface receptors were analyzed in accordance with the manufacturers' instructions. Briefly, phycoerythrin (PE)-conjugated mouse monoclonal anti-human LTβR antibody (R&D Systems Inc.) and fluorescein isothiocyanate (FITC)-labeled mouse monoclonal anti-human HVEM antibody (Medical & Biological Laboratories Co., Ltd., Nagoya, Japan) were used for detection of LTβR and HVEM, respectively, according to the manufacturers' instructions. Cells were run on an EPICS XL flow cytometer (Beckman Coulter Inc., Brea, CA) and analyzed using System II software.

### Cytokine array

The experiments were performed using the RayBio Human Cytokine Antibody Array 5 kit (AAH-CYT-5-x, RayBiotech Inc.) according to the manufacturer's instructions. The analyzed proteins included: angiogenin, BDNF, BLC, Ck-β8-1, EGF, ENA-78, eotaxin, eotaxin-2, eotaxin-3, FGF-4, FGF-6, FGF-7, FGF-9, Flt-3 ligand, fractalkine, G-CSF, GCP-2, GDNF, GM-CSF, GRO, GRO-α, HGF, I-309, IFN-γ, IGF-I, IGFBP-1, IGFBP-2, IGFBP-3, IGFBP-4, IL-1α, IL-1β, IL-2, IL-3, IL-4, IL-5, IL-6, IL-7, IL-8, IL-10, IL-12, IL-13, IL-15, IL-16, IP-10, leptin, LIF, LIGHT, MCP-1, MCP-2, MCP-3, MCP-4, MCSF, MDC, MIF, MIG, MIP-1β, MIP-1δ, MIP-3α, NAP-2, NT-3, NT-4, OSM, osteoprotegerin, PARC, PDGF-BB, PIGF, RANTES, SCF, SDF-1, TARC, TGF-β1, TGF-β2, TGF-β3, TIMP-1, TIMP-2, TNF-α, TNF-β, TPO and VEGF. Briefly, cell supernatants were put on the membranes for 2 h. After washing, array antibody and HRP-conjugated streptavidin were added to the membrane for 2 h. Membranes were detected with 1X detection buffer C and D, and pictures were taken with a cold CCD camera. Densitometric quantification of blots was performed using CS Analyzer 3.0 (ATTO). Different membranes were normalized using backgrounds.

### Quantitative reverse transcription-PCR (RT-PCR)

Total RNA was extracted from cells using an RNeasy Mini Kit (Qiagen, Tokyo, Japan). cDNA was synthesized using SuperScript III Reverse Transcriptase (Invitrogen, Carlsbad, CA) according to the manufacturer's protocol. Quantification of mRNA levels was performed using Mx-3000P (Stratagene, La Jolla, CA) and QuantiTect SYBR Green PCR (Qiagen) according to the manufacturers' instructions. Relative mRNA expression was calculated using the ΔΔCt method. Individual data were normalized against a housekeeping gene, glyceraldehyde-3-phosphate dehydrogenase (GAPDH). The specific primers for GAPDH, IL-8, LTβR, MCP-1, RANTES and IL-6 are shown in Table 1.

**Table 1:** Primer sequences.

Primer	Forward (5' to 3')	Reverse (5' to 3')
GAPDH	GGTGAAGGTCGGAGTCAACGCA	TCTTCCAGGAGGAGCGAGATCCCTG
IL-8	ACTGAGAGTGATTGAGAGTGGAC	AACCCTCTGCACCCAGTTTT
LTβR	GTTGAATCTGGCAGCCAAACC	ATGGAGGCACCTTTAATTGAGA
IL-6	ACTCACCTCTTCAGAACGAATTG	CCATCTTTGGAAGGTTCAGGTTG
MCP-1	CAGCCAGATGCAATCAATGCC	TGGAATCCTGAACCCACTTCT
RANTES	CGCTGTCATCCTCATTGCTA	ACACACTTGGCGGTTCTTTC

### Western blot analysis

Cells were lysed in a lysis buffer solution (20 mM Tris-HCl, 150 mM NaCl, 1 mM EDTA, 1% Nonidet P-40, 0.1% sodium deoxycholate, 0.1% SDS), followed by SDS gel-electrophoresis and semi-dry transfer of the proteins to polyvinylidene difluoride (PVDF) membranes. All sample protein concentrations were measured using the BCA Protein Assay Kit (Thermo Scientific, Waltham, MA), and the same amounts of protein were applied. Non-specific binding of proteins to the membranes was blocked by incubation in TBS-T buffer (50 mM Tris-HCl, pH 7.4, 150 mM NaCl and 0.1% Tween-20) with 2% ECL Prime Blocking Reagent (GE Healthcare, Buckinghamshire, UK), and the membranes were then incubated with primary antibodies. The antibodies and dilutions used in these studies are described below. Immunodetection was performed with the ECL Prime Western Blotting Detection Kit (GE Healthcare). Pictures were taken with a cold CCD camera (EZ-Capture MG; ATTO, Tokyo, Japan).

### Antibodies

The antibodies used were rabbit anti-p44/42 MAPK (Erk1/2) antibody #9102 (Cell Signaling Technology, Beverly, MA), rabbit anti-phospho-Erk1/2 antibody #9101 (Cell Signaling Technology), rabbit anti-phospho-p38 MAPK antibody #4511 (Cell Signaling Technology), rabbit anti-p38 MAPK antibody #8690 (Cell Signaling Technology), rabbit anti-phospho-SAPK/JNK antibody #4668 (Cell Signaling Technology), rabbit anti-SAPK/JNK antibody #9258 (Cell Signaling Technology), mouse anti-phospho-IκBα #9246 (Cell Signaling Technology), mouse anti-IκBα antibody #4814 (Cell Signaling Technology), rabbit anti-LTβR, N-Terminal antibody #SAB4501788 (Sigma-Aldrich), goat anti-LTβR antibody #L5412 (Sigma-Aldrich), normal goat IgG control #AB-108-C (R&D systems), anti-mouse-IgG HRP-linked antibody #7076 (Cell Signaling Technology), and anti-rabbit IgG-HRP-linked antibody #7074 (Cell Signaling Technology).

Equal protein loading was confirmed by probing the blot with mouse anti-α-tubulin (Sigma-Aldrich) antibody.

### Enzyme-linked immunosorbent assay (ELISA)

BEAS-2B and NHBE cells were seeded into each well of 24-well plates and stimulated with 50 ng/ml LIGHT for 24 h. IL-8 and IL-6 concentrations in the supernatant were measured using the PeliKine Compact human IL-8 ELISA kit and PeliKine Compact human IL-6 ELISA kit (Sanquin Blood Supply, Amsterdam, Netherlands) respectively, according to the manufacturer's instructions. The optical density was measured at 450 nm using a microplate reader (Bio-Rad, Hercules, CA). The concentrations were calculated using a standard curve obtained with the recombinant kit standards. The data were analyzed using the Microplate Manager 6 software (Bio-Rad).

### Transfection of small interfering RNA (siRNA)

All siRNAs were purchased from Invitrogen (Tokyo, Japan). Knockdown of LTβR was performed using two specific siRNA duplex (LTβR #1-#2) sets (Stealth RNAi Pre-Designed siRNAs). The sequences of the RNA duplexes are shown in Table 2. Stealth RNAi Negative Control Duplexes (Invitrogen) served as negative controls. Lipofectamine RNAiMAX Transfection Reagent (Invitrogen) was used for transfection in accordance with the manufacturer's instructions. Briefly, the cells were transfected with a final concentration of 10 nM of each siRNA duplex set. The cells were incubated for 24 h, and then the siRNA-containing medium was replaced with complete medium. 72 h after siRNA transfection, the cells were used for further experiments as “LTβR knocked-down” cells. The knockdown efficacy was confirmed by qRT-PCR and western blotting 72 h after transfection.

**Table 2:** siRNA sequences.

Set	Sense	Antisense
LTβR #1	UCUACAUCUACAAUGGACCAGUACU	AGUACUGGUCCAUUGUAGAUGUAGA
LTβR #2	UGCAAGGCAGGGCACUUCCAGAAUA	UAUUCUGGAAGUGCCCUGCCUUGCA

### Luciferase reporter assay

The transcription factor signal pathway from LTβR was determined using a pathway profiling system kit (#631911; BD Biosiences Clontech, UK). This kit was composed of several luciferase reporter vectors that contain a specific *cis*-acting DNA sequence (enhancer element) upstream of the luciferase gene and one construct (pTAL) without any enhancer element upstream of the luciferase reporter gene, used as a negative control. The key *cis*-acting elements tested in the study were: activator protein-1 (AP-1), nuclear factor of κB (NF-κB) and serum response element (SRE). All these specific *cis*-acting DNA binding sequences were located upstream of the TATA-like promoter (TAL) region from the herpes simplex virus thymidine kinase (HSV-TK) promoter. The vector pTAL was used as a null vector, which did not have any *cis*-acting elements in its promoter region and was a negative control in the assay. To normalize the transfection efficiency, cells were co-transfected with pRL-TK *Renilla* luciferase (pRL-TK-Rluc). The vectors were transfected into BEAS-2B cells using FuGENE HD transfection reagent (Promega, Tokyo, Japan) according to the manufacturer's protocol. Briefly, BEAS-2B cells were transfected with 0.5 µg of the reporter vector and 0.05 µg of pRL-TK-Rluc. The cell supernatant was replaced by fresh medium 5 h after transfection. The cells were stimulated with 50 ng/ml LIGHT 24 h after transfection and collected 24 h later. Luciferase activities were measured with the Dual-Luciferase Reporter Assay System (Promega) using a luminometer (Luminescencer-Octa, AB-2270; ATTO). The relative luciferase activity was examined in triplicate and normalized to *Renilla* luciferase activity.

### Statistical analysis

Results were confirmed by repeating experiments on at least three separate occasions. Data shown in the figures are pooled data for each experiment and expressed as the mean ± SEM. Analyses were performed using JMP Pro (Version 11.1.1; SAS Institute Japan Ltd., Tokyo, Japan). Samples with multiple comparisons were analyzed for significance by analysis of variance (ANOVA). When ANOVA indicated a significant difference between groups, Tukey-Kramer's HSD was applied. P values of <0.05 were considered to be significant.

## Results

### LTβR and HVEM expression by bronchial epithelial cells

**Figure 1:**
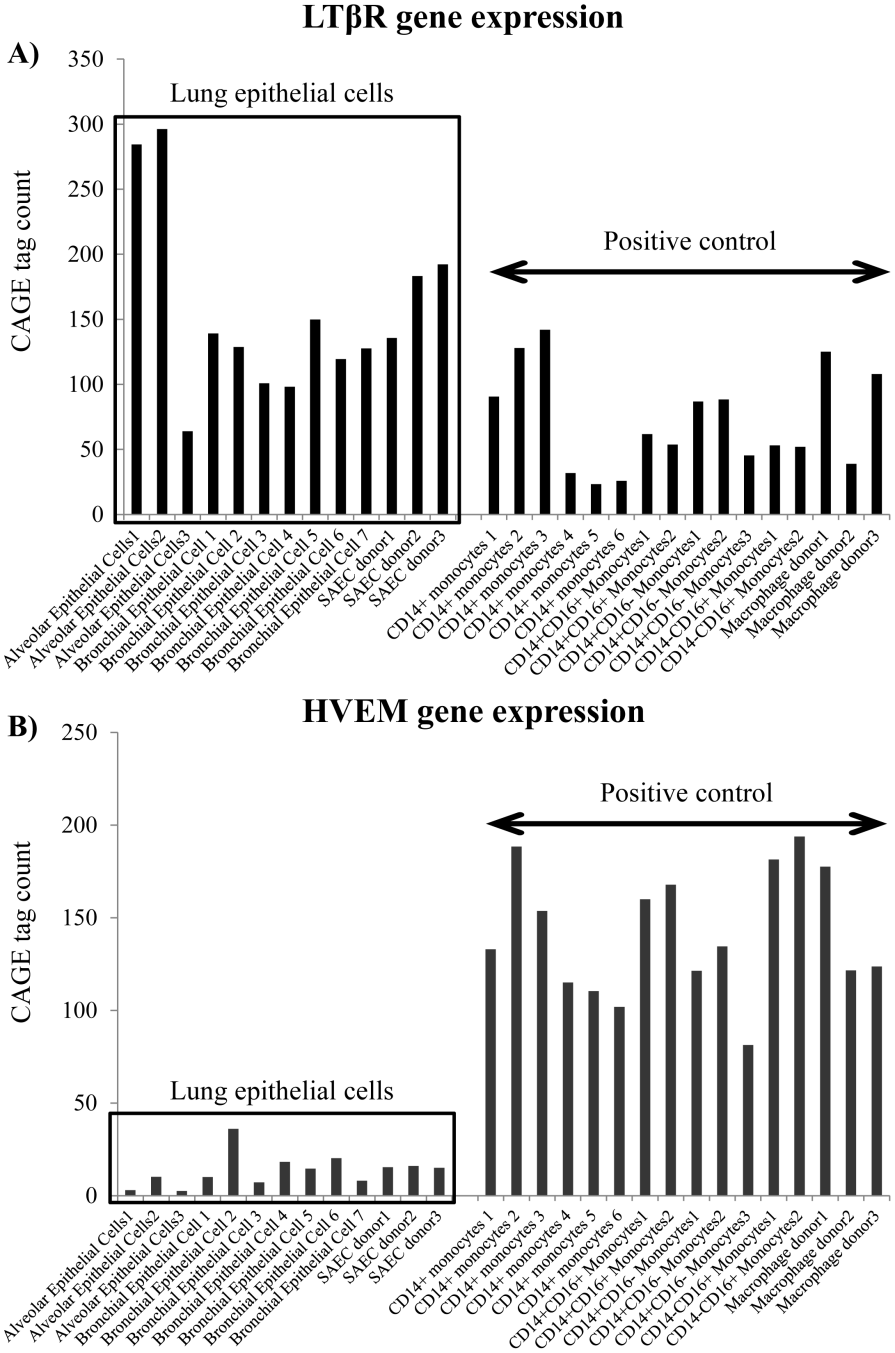
Gene expression analysis of primary lung epithelial cells. We analyzed gene expression of LTβR and HVEM in several types of primary cells using the ZENBU database, which analyzed CAGE (cap analysis gene expression) of 432 normal primary cells. The lung epithelial cells strongly expressed the LTβR gene (A), but not the HVEM gene (B).ZENBU database URL: http://fantom.gsc.riken.jp/zenbu/

**Figure 2:**
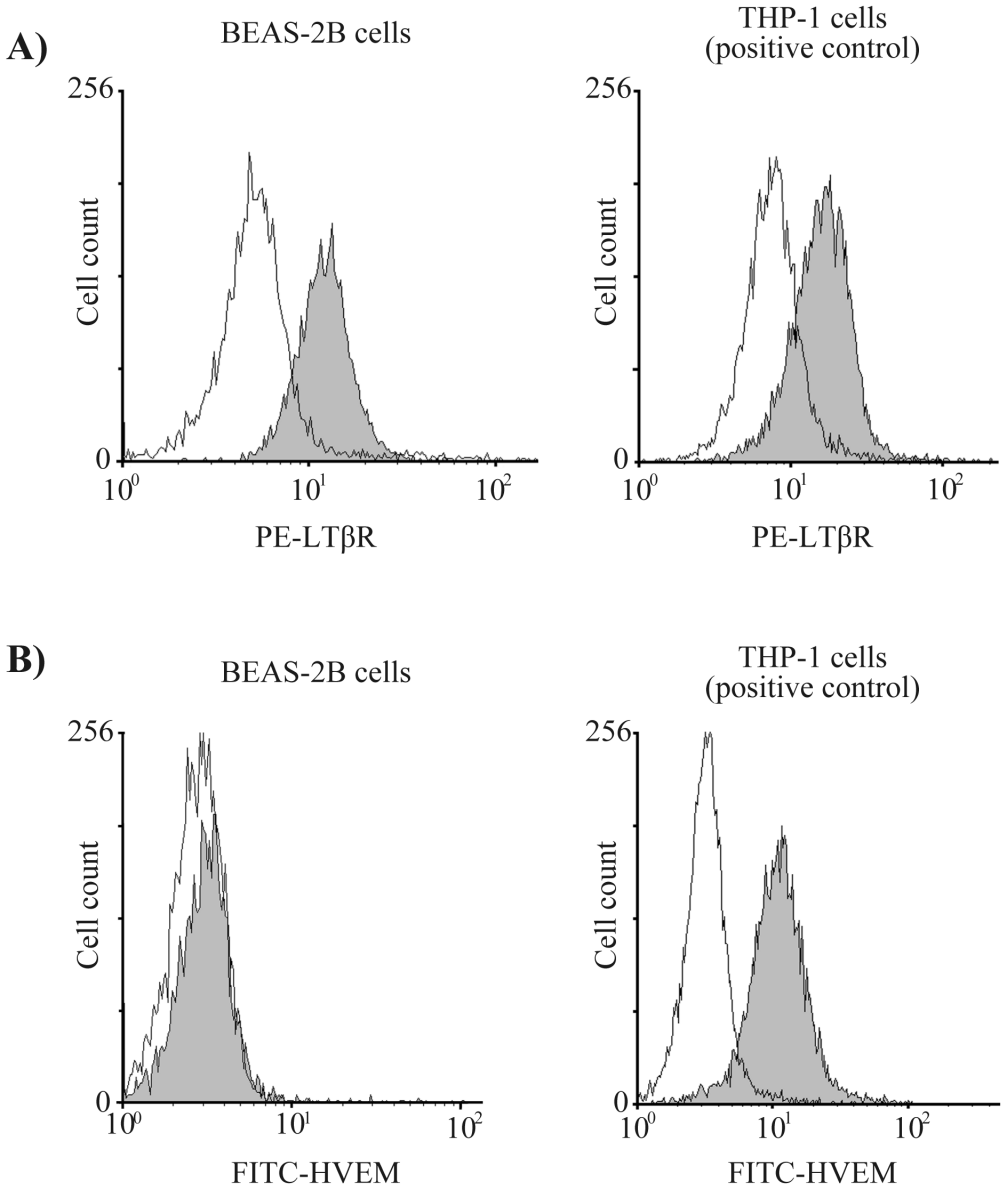
Expression of LTβR and HVEM receptors on BEAS-2B cells. BEAS-2B cells and THP-1 cells (positive control) were treated with anti-PE labeled LTβR antibody and anti-FITC labeled HVEM antibody. The fluorescence intensity was measured with a flow cytometer. THP-1 cells, a human monocytic cell line, were used as the positive control because they express both LTβR and HVEM on their cell surface. The fluorescence intensities of LTβR (A) and HVEM (B) suggest that BEAS-2B cells express LTβR but not HVEM.

We first evaluated gene expression of LTβR and HVEM in various types of primary cells using the “ZENBU” database of CAGE (cap analysis gene expression) results for 432 normal primary cells [22]. The bronchial epithelial cells expressed the LTβR gene strongly, but the HVEM gene less so (Fig. 1). We also examined the receptors' expression on the bronchial epithelial cell surface by flow cytometry, and the results were the same as those for the “ZENBU” database (strong LTβR expression, weaker HEVM expression) (Fig. 2).

### Comprehensive analysis of chemokine production by BEAS-2B cells

**Figure 3:**
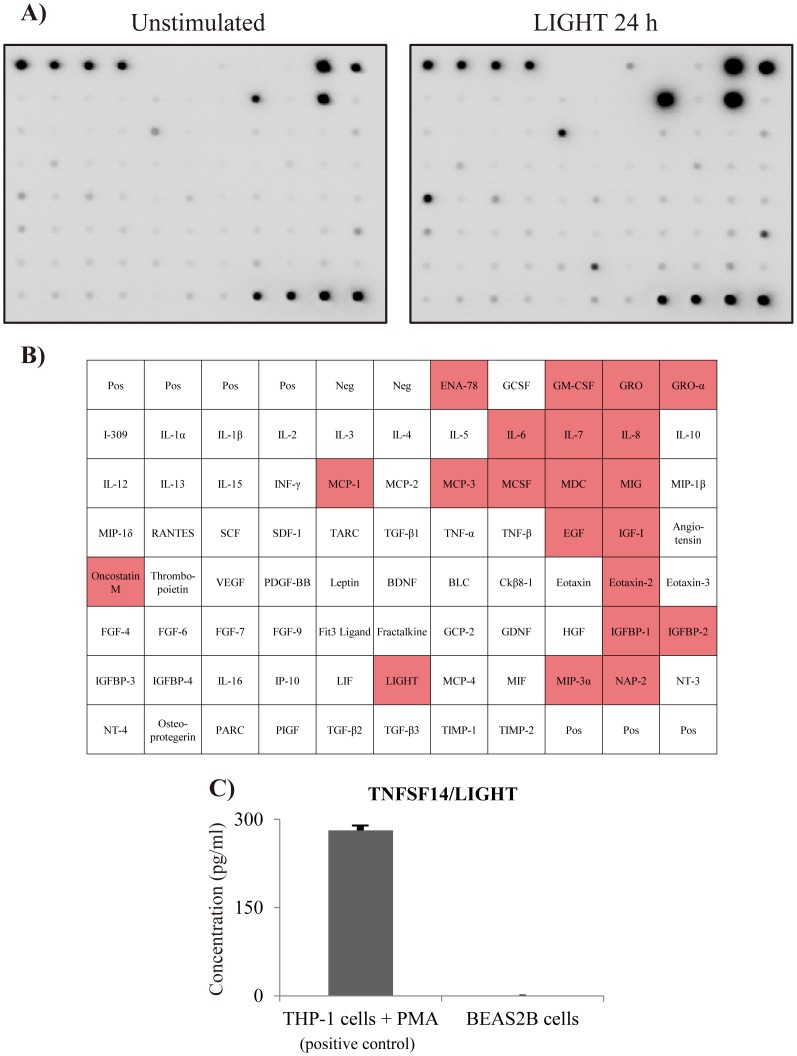
Comprehensive analysis of LIGHT-induced cytokine and chemokine production. BEAS-2B cells were stimulated with LIGHT (100 ng/ml) for 24 h, followed by determination of the protein levels of cytokines and chemokines by densitometry using a cytokine array. (A) The left image shows the unstimulated samples, while the right image shows the samples at 24 h after stimulation with LIGHT. (B) This table shows array mapping. The red color indicates cytokines that were upregulated more than twofold compared to the unstimulated sample. LIGHT induced inflammatory cytokines, such as GRO, GRO-α, oncostatin M, MCP-1, IL-6 and IL-8. (C) We investigated whether BEAS-2B cells produced LIGHT. THP-1 cells, which were used as a positive control, produced LIGHT when stimulated with PMA 50 ng/ml, but BEAS-2B cells did not.

We used a cytokine array that detects 79 cytokines in order to analyze cytokines and chemokines secreted by bronchial epithelial cells (BEAS-2B) stimulated with LIGHT. As shown in Fig. 3B, LIGHT induced various cytokines, such as GRO, GRO-α, oncostatin M and MCP-1, and especially IL-6 and IL-8. These results indicate that LIGHT is a potent inducer of inflammatory cytokine and chemokine production by bronchial epithelial cells. We also demonstrated that BEAS-2B cells themselves did not produce LIGHT (Fig. 3C).

### IL-8 and IL-6 expression by bronchial epithelial cells induced by LIGHT

**Figure 4:**
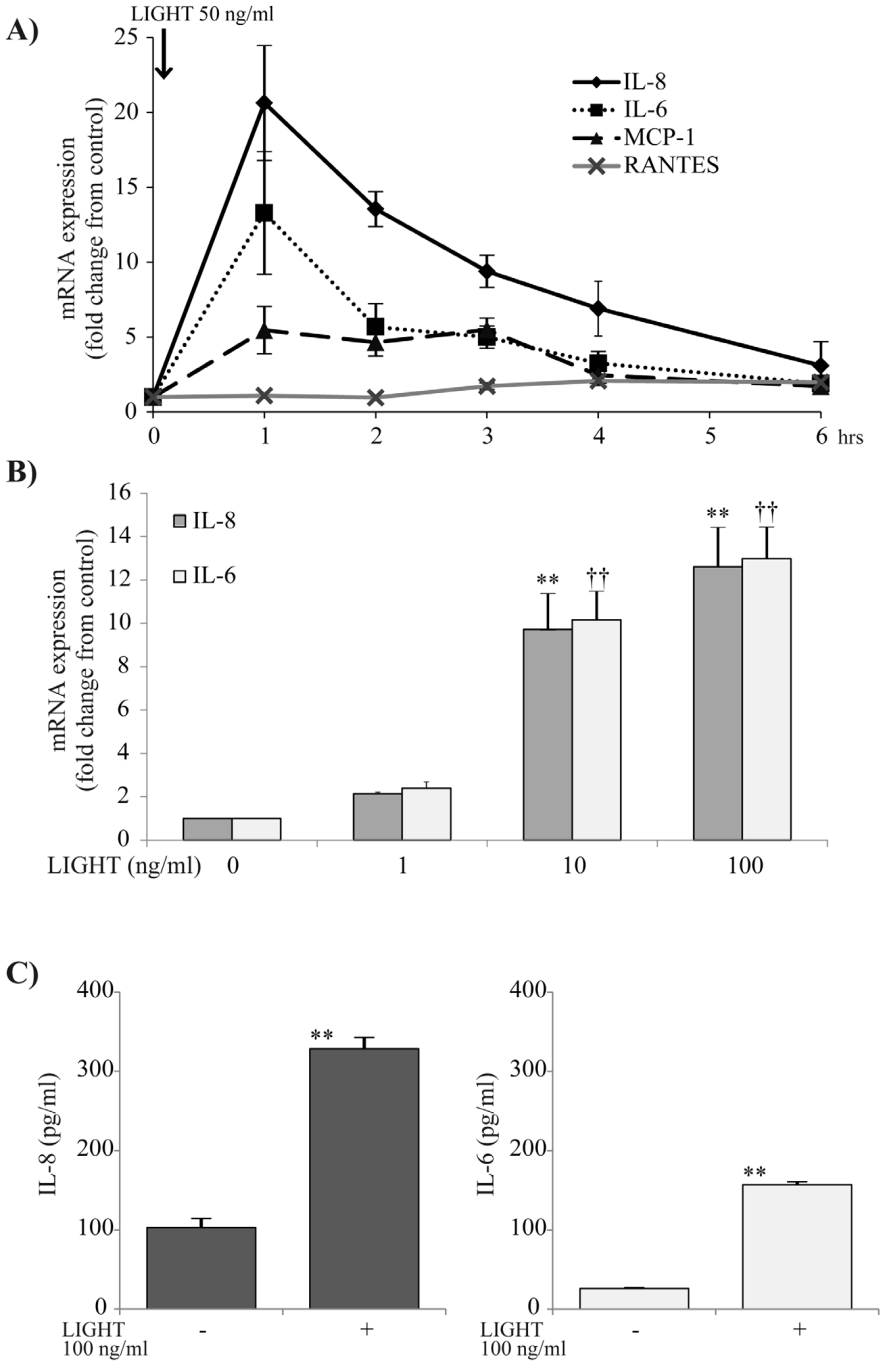
Time-course and dose-dependent effects of LIGHT on bronchial epithelial (BEAS-2B) cells. (A) BEAS-2B cells were stimulated with 50 ng/ml LIGHT and examined for the time-course effect on expression of mRNA for each of IL-8, IL-6, MCP-1 and RANTES. LIGHT significantly induced mRNA for each of IL-6, IL-8 and MCP-1. n = 4 separate experiments. *: p<0.05, **: p<0.01 vs 0 h. (B) BEAS-2B cells were stimulated with various concentrations of LIGHT (0, 1, 10, 100 ng/ml) for 1 h and evaluated for expression of mRNA for each of IL-8 and IL-6. LIGHT induced IL-8 and IL-6 mRNA dose-dependently. n = 4 separate experiments. ** and ††: p<0.01 vs 0 ng/ml. (C) BEAS-2B cells were stimulated with 100 ng/ml LIGHT for 24 h, and the protein concentrations in the cell supernatants were determined by ELISA. LIGHT significantly induced IL-8 and IL-6 proteins. n = 6 separate experiments. **: p<0.01.

To determine the effect of LIGHT on BEAS-2B cells, we analyzed the time-course effect on expression of mRNA for several inflammatory chemokines. As shown in Fig. 4A, LIGHT significantly induced IL-8, IL-6 and MCP-1 mRNA at 1 h after stimulation. Then the cells were stimulated with several concentrations of LIGHT, and IL-8 and IL-6 mRNA expression was measured after 1 h. LIGHT induced IL-8 and IL-6 mRNA dose-dependently (Fig. 4B). In addition, as shown in Fig. 4C, LIGHT had significantly induced IL-8 and IL-6 proteins in BEAS-2B cells at 24 h after stimulation. We also confirmed by MTT assay that LIGHT, at less than 100 ng/ml, did not show any cytotoxic effect on BEAS-2B cells (data not shown). As shown in Fig. 4A, LIGHT induced clear IL-8 production at a concentration of 50 ng/ml. In consideration of that result, we used LIGHT at that concentration for subsequent experiments.

### Involvement of LTβR in LIGHT-induced IL-8 production

**Figure 5:**
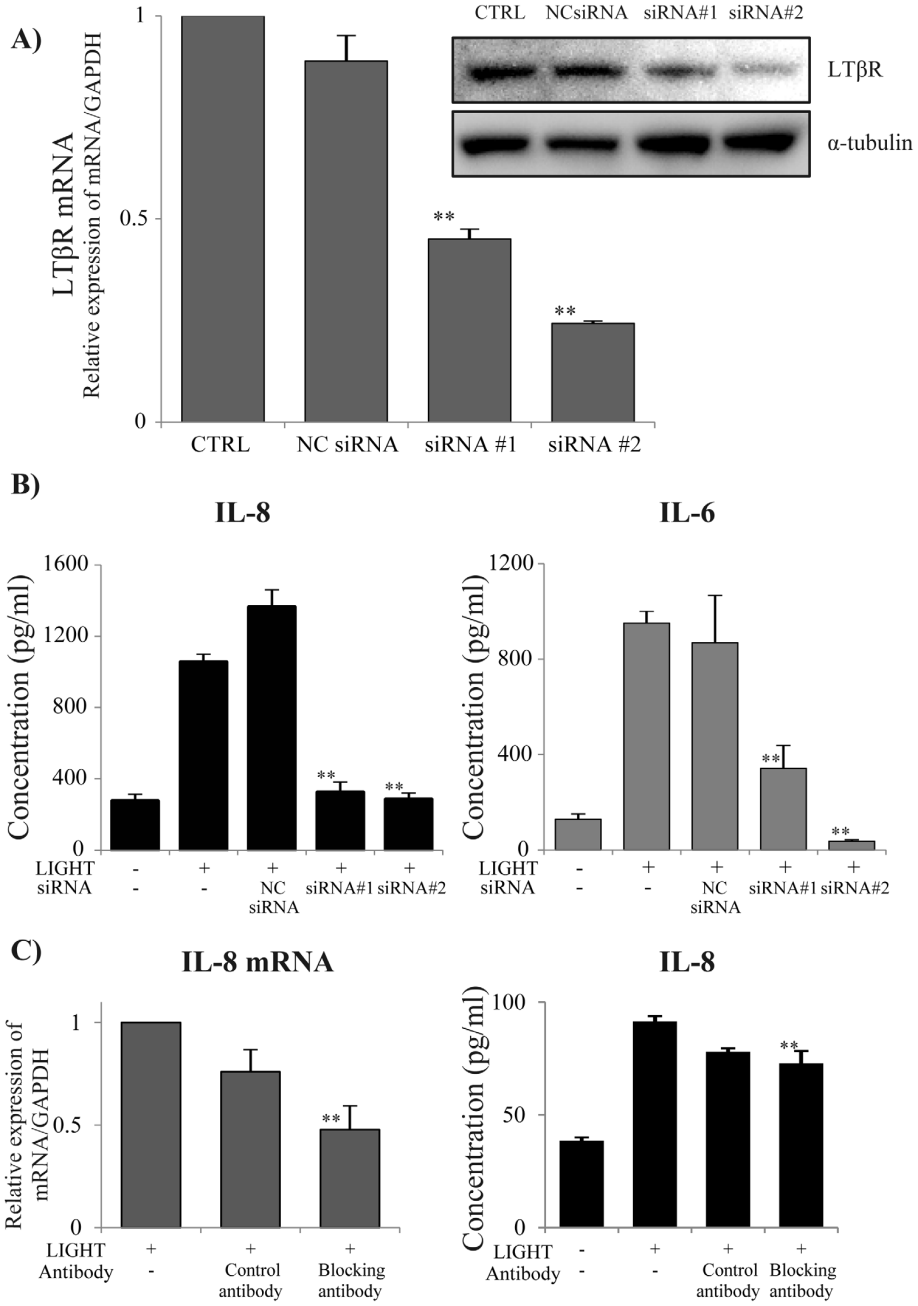
Effect of LTβR siRNA on IL-8 production by BEAS-2B cells. BEAS-2B cells were transfected with LTβR siRNA to knock down the receptor. (A) We purchased two types of siRNA (#1 and #2) and transfected them into BEAS-2B cells. We evaluated the knockdown efficacy of each siRNA 72 h later by qRT-PCR and western blotting. Lipofectamine reagent and the negative control siRNA (NC siRNA) did not affect LTβR mRNA expression, but siRNA#1 and #2 both significantly inhibited LTβR mRNA. (B) BEAS-2B cells were transfected with siRNA, and 72 h later they were stimulated with 50 ng/ml LIGHT. The IL-8 and IL-6 concentrations in the cell supernatants were determined by ELISA 24 h after stimulation. Both siRNA#1 and siRNA#2 significantly inhibited IL-8 and IL-6 production by BEAS-2B cells. n = 4 separate experiments. **: p<0.01. (C) BEAS-2B cells were pre-incubated with LTβR blocking antibody before stimulation with 50 ng/ml LIGHT. The blocking antibody for LTβR significantly attenuated both IL-8 mRNA expression and IL-8 production. n = 3 separate experiments. **: p<0.01.

We used siRNA for LTβR to evaluate whether IL-8 production by BEAS-2B cells was induced by LTβR signaling. As shown in Fig. 5A, we found that siRNA #1 and #2 strongly inhibited LTβR expression. siRNA #1 and #2 also strongly inhibited IL-8 production by cells that were stimulated with LIGHT (Fig. 5B). In consideration of that result, we used siRNA #2 for subsequent experiments. These results indicated that LTβR signaling is important for LIGHT-induced cytokine production by BEAS-2B cells. We also evaluated the effect of blocking antibody for LTβR. As shown in Fig. 5C, the blocking antibody for LTβR significantly suppressed LIGHT-induced IL-8 mRNA and inhibited LIGHT-induced IL-8 production.

### LTβR signaling in bronchial epithelial cells induced by LIGHT

**Figure 6:**
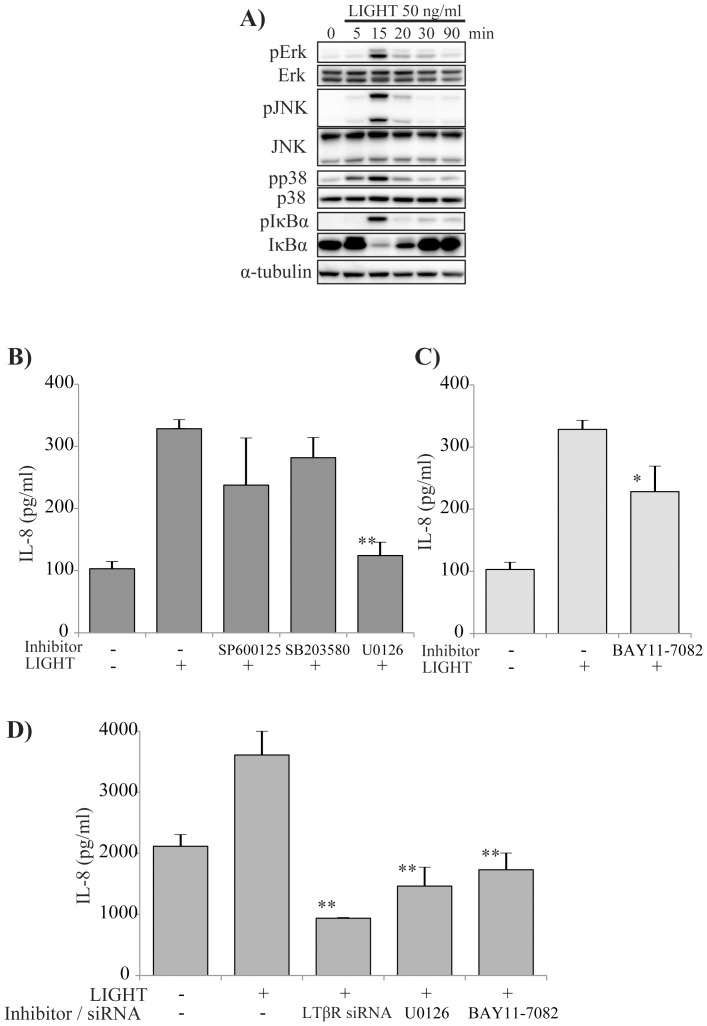
LTβR signaling in bronchial epithelial cells induced by LIGHT. BEAS-2B cells were stimulated with 50 ng/ml LIGHT, and cell lysates were prepared 0, 5, 15, 20, 30 and 90 minutes later to evaluate phosphorylation of Erk, JNK, p38 and IκB. The MAPKs were phosphorylated for up to 15 minutes, and their signaling was activated. IκB was also phosphorylated at the same time and induced NF-κB release and translocation to nucleus. (A) BEAS-2B cells were pre-treated for 1 h with various inhibitors of phosphorylation of MAPKs. U0126 significantly inhibited IL-8 production by the cells. (B) BEAS-2B cells were pre-treated for 1 h with BAY11-7082. BAY11-7082 significantly inhibited IL-8 production. (C) We performed the same experiments using NHBE cells, which are primary normal human bronchial epithelial cells. LTβR siRNA, U0126 and BAY11-7082 significantly inhibited IL-8 production by NHBE cells in the same manner as seen for BEAS-2B cells.

In order to determine the mechanism of LIGHT-induced IL-8 production by bronchial epithelial cells, we evaluated MAPKs signaling and NF-κB signaling. As shown in Fig. 6A, LIGHT potently induced MAPKs signals, and also IκBα phosphorylation. Moreover, to examine whether induction of MAPKs and NF-kB signaling contributed to production of IL-8, we used specific inhibitors of signaling. U0126, but not SP600125 or SB203580, significantly inhibited IL-8 production (Fig. 6B). These results suggest that LIGHT can induce Erk1/2, p38 and JNK signaling, but the major contributor to IL-8 production is Erk1/2 signaling. BAY11-7082 also inhibited IL-8 production by the cells (Fig. 6C). We also examined the mechanism of IL-8 production by NHBE cells. As shown in Fig. 6D, U0126, BAY11-7082 and siRNA for LTβR each inhibited IL-8 production by NHBE cells, similar to BEAS-2B cells.

### Erk1/2 signaling and NF-κB release

**Figure 7:**
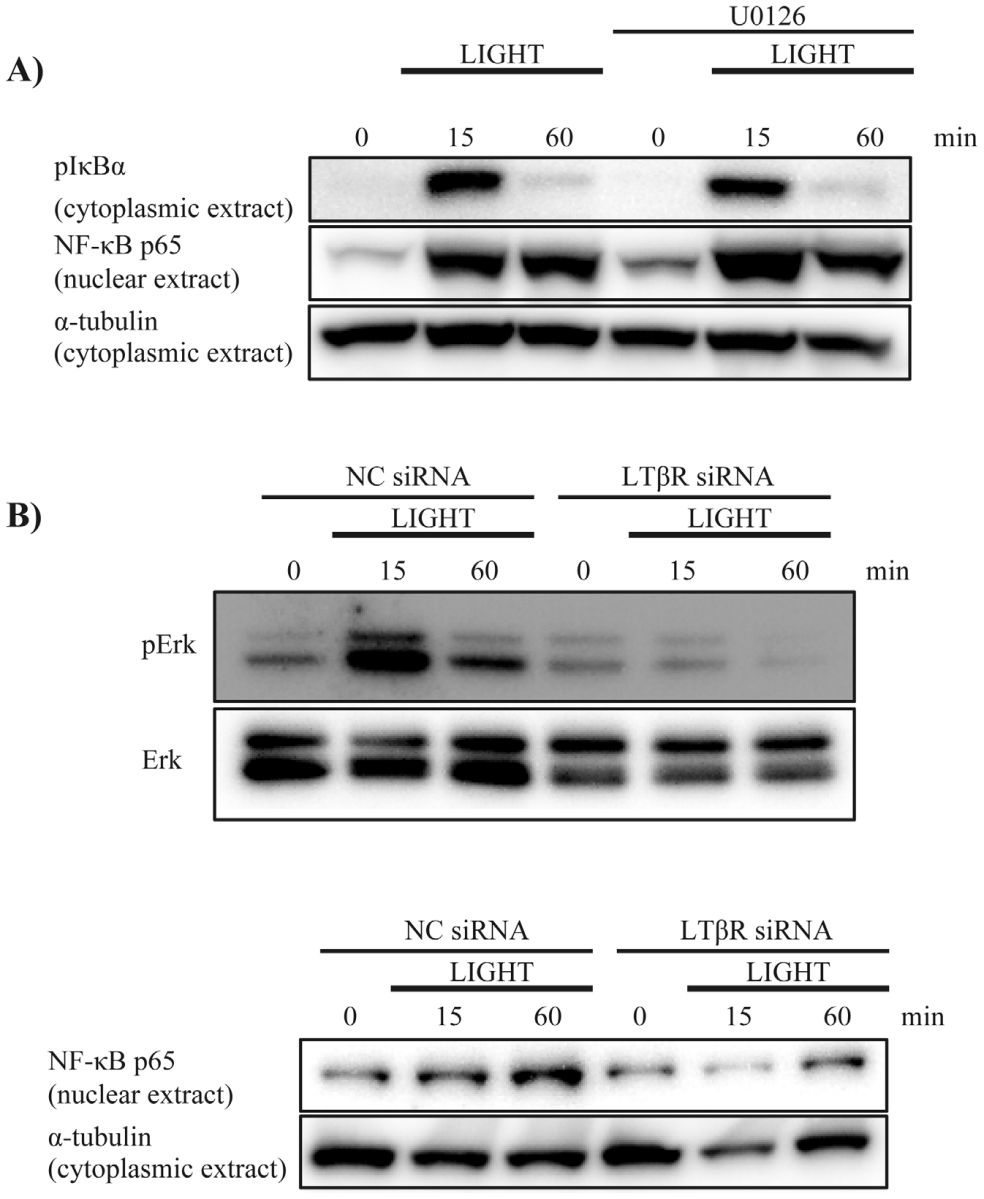
Erk1/2 signaling and NF-κB release. (A) To evaluate the relationship between Erk1/2 signaling and NF-κB release, BEAS-2B cells were pretreated with U0126 (10 µM) 1 h before stimulation with LIGHT. U0126 did not inhibit IκBα phosphorylation or NF-κB translocation from the cytoplasm to the nucleus. (B) We evaluated the effect of LTβR knockdown on Erk1/2 signaling and NF-κB release. BEAS-2B cells were transfected with negative control siRNA (NC siRNA) or LTβR siRNA#2, stimulated with LIGHT (50 ng/ml) and analyzed by western blotting. The cells that were transfected with LTβR siRNA showed attenuation of Erk1/2 phosphorylation and NF-κB translocation induced by LIGHT.

**Figure 8:**
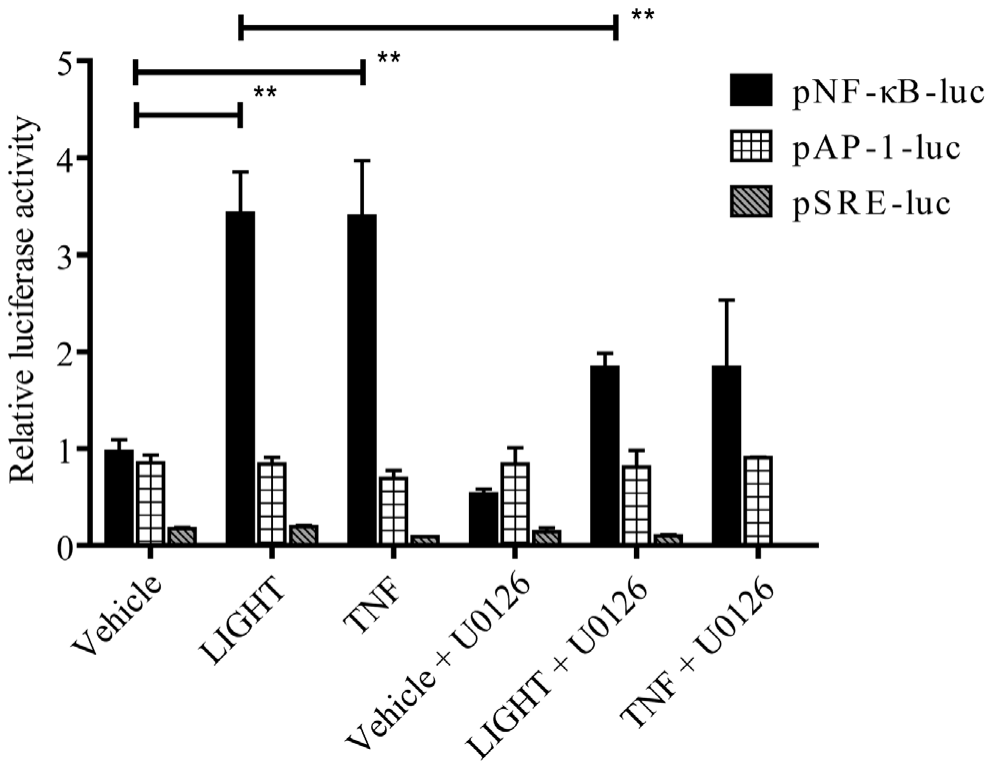
Effect of LTβR signaling on transcription factor-driven luciferase activity by transient transfection of BEAS-2B cells. BEAS-2B cells were transfected with a plasmid having luciferase as a reporter gene, controlled by a synthetic promoter containing NF-κB, AP-1 or serum response element (SRE). After 24 h, the cells were stimulated with LIGHT (50 ng/ml) or TNF-α (10 ng/ml) as a positive control, with/without U0126. The data are the mean ± SEM of four independent experiments performed in triplicate. Significant differences (p<0.01) are indicated with **.

Based on the above results, we examined whether Erk1/2 signaling could induce NF-κB for IL-8 production. U0126 did not inhibit IκBα phosphorylation or NF-κB translocation to the nucleus (Fig. 7A). In contrast, LTβR siRNA attenuated Erk1/2 phosphorylation and NF-κB translocation (Fig. 7B). These results indicate that Erk1/2 signaling and NF-κB signaling are down-stream of LTβR signaling, but NF-κB signaling is not directly down-stream of Erk1/2 signaling. We also performed luciferase reporter assay to evaluate whether LTβR signaling could activate transcription factor response elements. As shown in Fig. 8, LIGHT induced luciferase activity of NF-κB response element, but not AP-1 or SRE. U0126 partially attenuated the luciferase activity of NF-κB response element induced by LIGHT, indicating that Erk1/2 signaling is partially involved in induction of NF-κB expression.

## Discussion

We demonstrated that LIGHT induced several inflammatory cytokines and chemokines, including IL-8, in BEAS-2B and NHBE cells. Human bronchial epithelial cells expressed LTβR, a signal that induces the Erk and NF-κB pathways for IL-8 production.

IL-8/C-X-C motif ligand (CXCL) 8 is produced by epithelial cells, airway smooth muscle cells and endothelial cells. It is a member of the α-CXC chemokine family. IL-8 is an inflammatory mediator that induces chemotaxis of neutrophils and other granulocytes, cells that release reactive oxygen species and elastase. Indeed, the number of neutrophils in sputum is higher in severe asthma compared to mild and moderate asthma [5]. Moreover, IL-8 is upregulated in the airway of severe asthmatics [23] and contributes to prolongation of steroid-resistant inflammation via accumulation of neutrophils [24], [25]. IL-8 binds to G protein–coupled chemokine receptors (CXCR) 1 and CXCR2, which promote neutrophil influx into tissue sites of inflammation and induce acute lung injury [26]. In consideration of this background, IL-8 may play a key role in chronic airway inflammation in severe asthma through induction of neutrophilic inflammation [27].

We hypothesized that LIGHT and LTβR signaling contribute to severe asthma through induction of IL-8, which promotes accumulation of neutrophils and inflammation in the airway. First, we demonstrated cytokine production, especially IL-8, by BEAS-2B cells and NHBE cells when they were stimulated with LIGHT.

We examined expression of receptors for LIGHT using flow cytometry. It is known that the functional receptors of LIGHT are LTβR and HVEM [28]. LTβR is highly expressed in the lung, liver and kidney, and moderately expressed in the heart and testes. It is weakly expressed in the brain, thymus, spleen and lymph nodes [29]. On the other hand, HVEM expression is high in naïve and memory B cells, but it is not present on activated B cells in the germinal center [30], [31]. Here, we found that BEAS-2B cells and NHBE cells expressed only LTβR. Based on that result, LTβR and its downstream signaling were considered as key players in cytokine production by bronchial epithelial cells. So we evaluated the profile of LTβR signaling-induced cytokines using not only a cytokine array but also qRT-PCR or ELISA. We showed that LIGHT induced various inflammatory cytokines, such as GRO, GRO-α, oncostatin M, MCP-1, IL-6 and IL-8, but not RANTES. IL-6 and IL-8 were induced dose-dependently.

Next, we analyzed the mechanism of cytokine production by the cells. Because previous studies showed MAPKs to be involved not only in TNF-α-induced IL-8 production [32], but also in steroid-resistant inflammation in severe asthma [33], we focused on this signaling. LIGHT induced not only MAPKs signaling, such as Erk1/2, p38 and JNK, but also NF-κB signaling. We used specific phosphorylation inhibitors to determine which pathways were most important for IL-8 production. U0126 and BAY11-7082—a MEK1/2 inhibitor and an IκBα phosphorylation inhibitor, respectively—both inhibited IL-8 production. On the other hand, SP600125 and SB20358—JNK and p38 phosphorylation inhibitors—did not attenuate IL-8 production. In addition, we used a specific siRNA for LTβR, and it significantly attenuated IL-8 production and simultaneously attenuated Erk1/2 and IκBα phosphorylation. These results indicate that LIGHT-induced IL-8 production is due to activation of Erk1/2 and NF-κB via LTβR.

**Figure 9:**
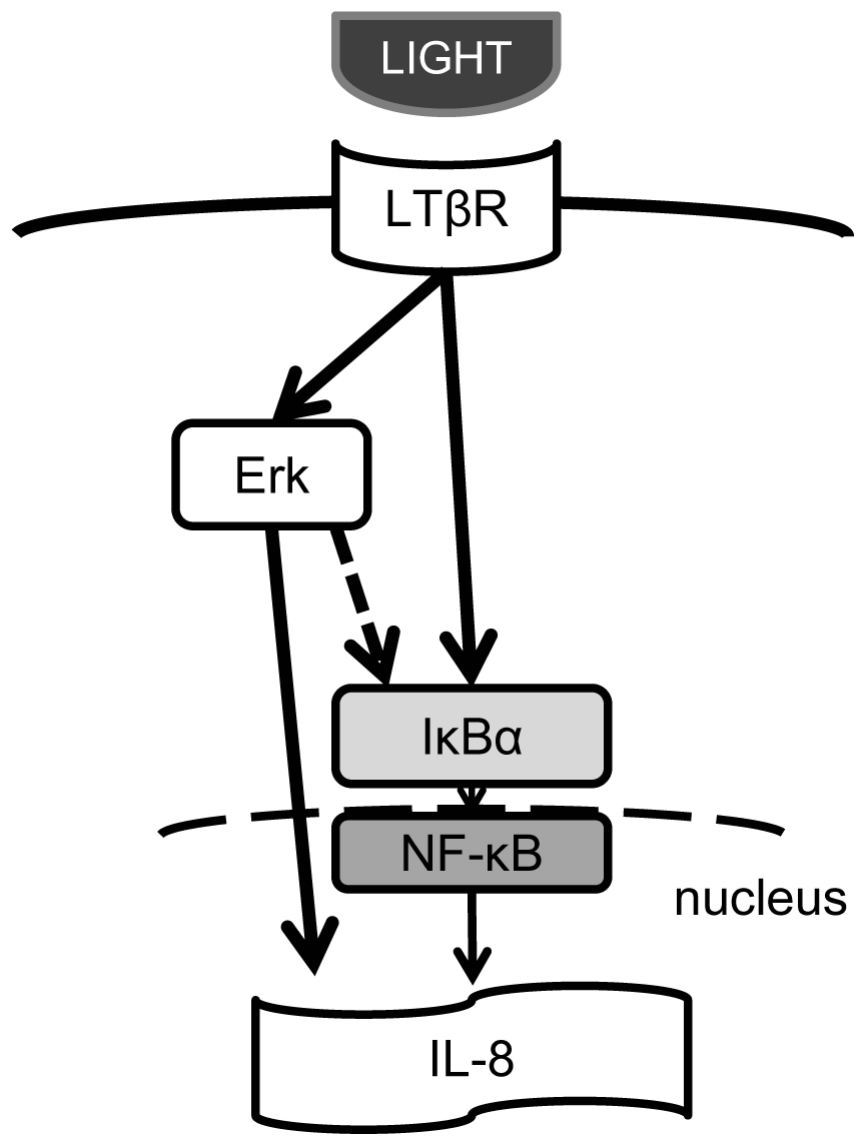
Schematic summary of how LTβR signal transduction induces IL-8 expression in human bronchial epithelial cells. LIGHT binding to LTβR may induce Erk1/2 or IκBα phosphorylation, which in turn induces NF-κB activation, and ultimately causes IL-8 release from the cells.

Some transcriptional factors, such as NF-κB and AP-1, were reported to regulate IL-8 expression [34]. It is thought that LIGHT induces IL-8 production through NF-κB release [35], [36]. Our results showed that U0126 completely inhibited IL-8 production, so we investigated the possibility that the Erk1/2 pathway activated the NF-κB pathway. Indeed, U0126 partially attenuated LIGHT-induced luciferase activity of NF-κB response element, but it did not inhibit NF-κB release and translocation to the nucleus. As shown in Fig. 9, these results suggested that there were two pathways by which LIGHT induced IL-8: (1) via Erk1/2 and (2) directly via NF-κB. Although the mechanism by which Erk1/2 induces IL-8 gene expression is not clear, Erk2 (p44/42 MAPK) was reported to phosphorylate Elk-1 and activates SRE, leading to induction of *c-fos*, a component of AP-1 [37]. The AP-1 binding element is present in the promoter region of the IL-8 gene, and AP-1, a complex of *c-fos* and *c-jun* or *jun* B, plays an important role in transcriptional regulation of IL-8 mRNA expression [38]–[40]. On the other hand, it has been considered that LIGHT induces two types of NF-κB pathway. One is degradation of IκBα to release p50/RelA and p50/c-Rel heterodimers via TNF receptor-associated factors [41]. The other is involvement of LTβR in NF-κB-inducing kinase activation to promote p100 processing [42].

As described above, LTβR is a key receptor involved in airway inflammation through LIGHT-induced cytokine production. Blockade of LTβR by administering specific antibodies or siRNA might reduce excessive cytokine production. This might be a new therapeutic approach for, for example, severe asthma patients with a high sputum level of LIGHT. A limitation of this study is that we did not test our *in vitro* findings by performing *in vivo* experiments, such as in a murine asthma model. Such studies will be needed.

## Conclusion

LIGHT, via LTβR signaling, may contribute to exacerbation of airway inflammation through cytokine and chemokine production by bronchial epithelial cells. The mechanism of LIGHT-induced cytokine production, especially for IL-8, is activation of LTβR signaling that activates the Erk and NF-κB pathways. Since LTβR is expressed on various airway epithelial cells, the LTβR signaling pathway might represent a new therapeutic target for severe asthma.
